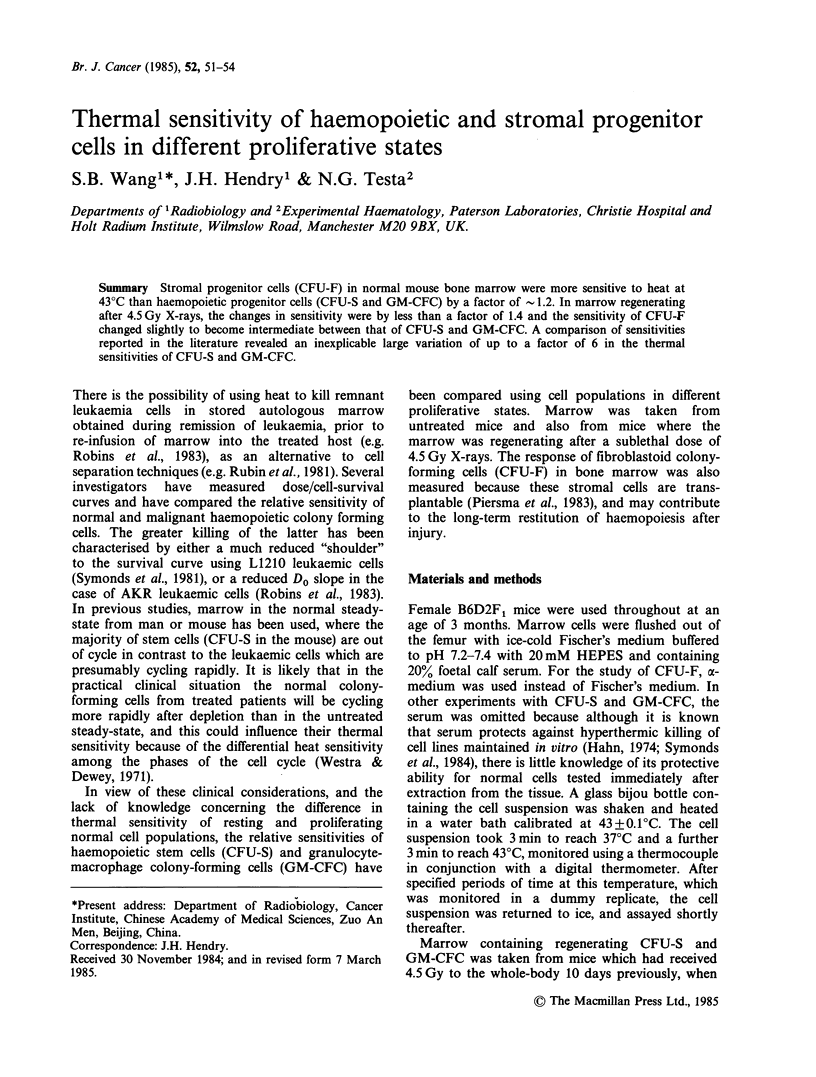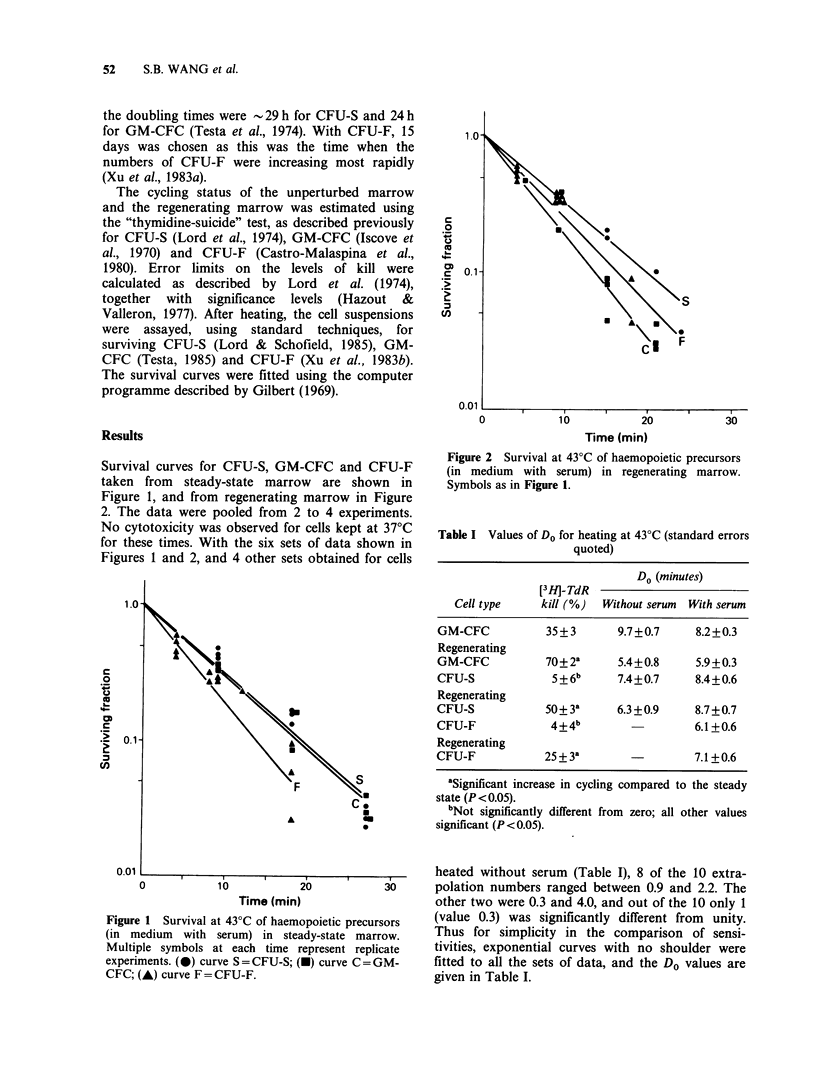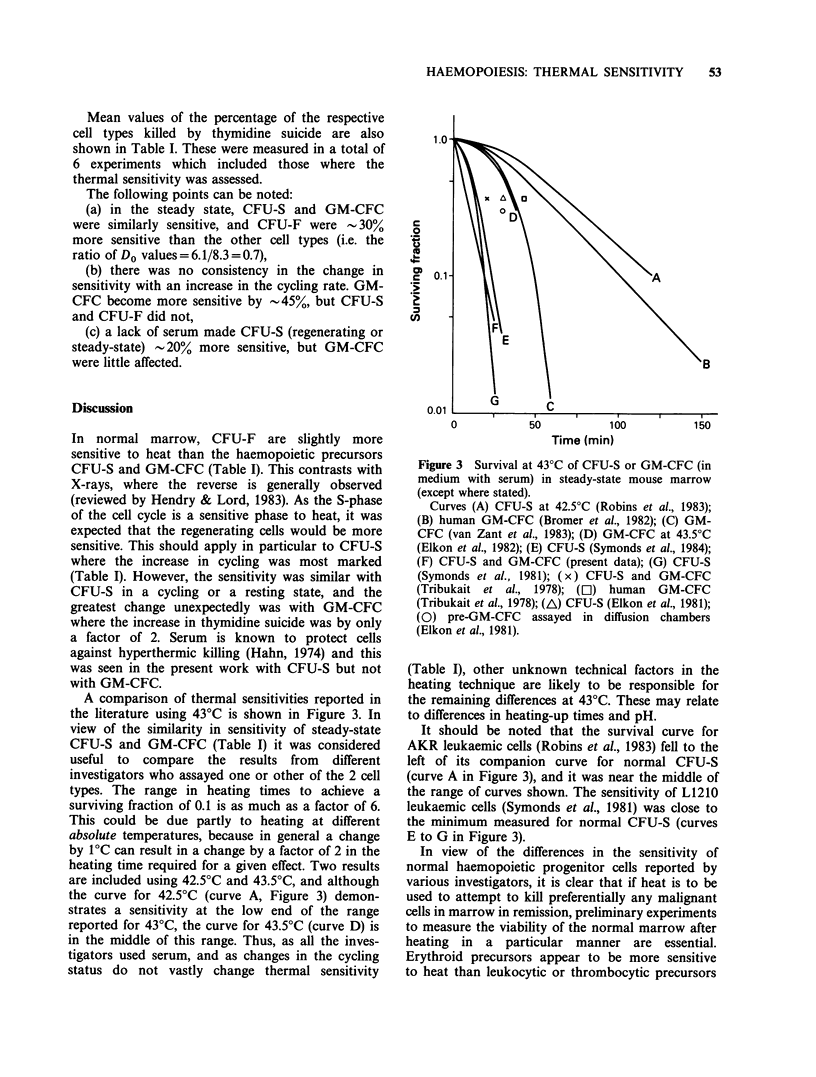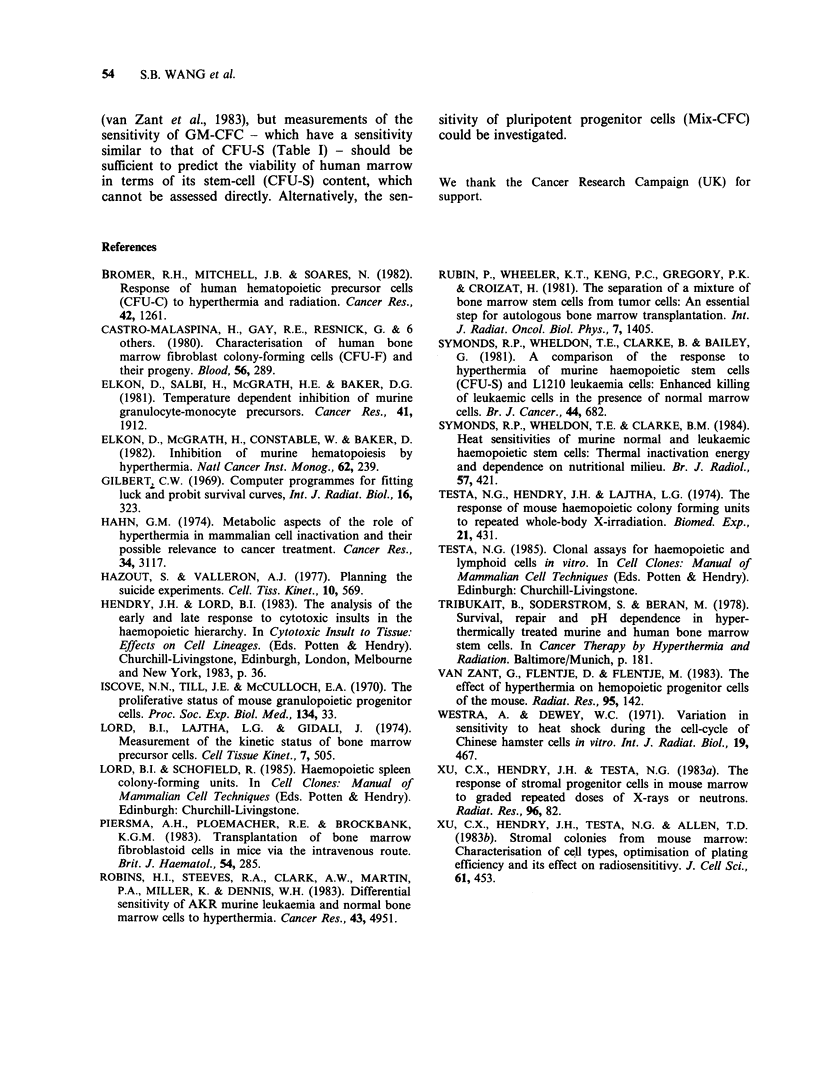# Thermal sensitivity of haemopoietic and stromal progenitor cells in different proliferative states.

**DOI:** 10.1038/bjc.1985.147

**Published:** 1985-07

**Authors:** S. B. Wang, J. H. Hendry, N. G. Testa

## Abstract

Stromal progenitor cells (CFU-F) in normal mouse bone marrow were more sensitive to heat at 43 degrees C than haemopoietic progenitor cells (CFU-S and GM-CFC) by a factor of approximately 1.2. In marrow regenerating after 4.5 Gy X-rays, the changes in sensitivity were by less than a factor of 1.4 and the sensitivity of CFU-F changed slightly to become intermediate between that of CFU-S and GM-CFC. A comparison of sensitivities reported in the literature revealed an inexplicable large variation of up to a factor of 6 in the thermal sensitivities of CFU-S and GM-CFC.


*Br. J. Cancer* (1985), **52**, 51–54

### Thermal sensitivity of haemopoietic and stromal progenitor cells in different proliferative states

S.B. Wang[1]*, J.H. Hendry[1] & N.G. Testa[2]

*Departments of [1]Radiobiology and [2]Experimental Haematology, Paterson Laboratories, Christie Hospital and Holt Radium Institute, Wilmslow Road, Manchester M20 9BX, UK.*

**Summary** Stromal progenitor cells (CFU-F) in normal mouse bone marrow were more sensitive to heat at 43°C than haemopoietic progenitor cells (CFU-S and GM-CFC) by a factor of ~1.2. In marrow regenerating after 4.5 Gy X-rays, the changes in sensitivity were by less than a factor of 1.4 and the sensitivity of CFU-F changed slightly to become intermediate between that of CFU-S and GM-CFC. A comparison of sensitivities reported in the literature revealed an inexplicable large variation of up to a factor of 6 in the thermal sensitivities of CFU-S and GM-CFC.

There is the possibility of using heat to kill remnant leukaemia cells in stored autologous marrow obtained during remission of leukaemia, prior to re-infusion of marrow into the treated host (e.g. Robins *et al.*, 1983), as an alternative to cell separation techniques (e.g. Rubin *et al.*, 1981). Several investigators have measured dose/cell-survival curves and have compared the relative sensitivity of normal and malignant haemopoietic colony forming cells. The greater killing of the latter has been characterised by either a much reduced "shoulder" to the survival curve using L1210 leukaemic cells (Symonds *et al.*, 1981), or a reduced $D_0$ slope in the case of AKR leukaemic cells (Robins *et al.*, 1983). In previous studies, marrow in the normal steady-state from man or mouse has been used, where the majority of stem cells (CFU-S in the mouse) are out of cycle in contrast to the leukaemic cells which are presumably cycling rapidly. It is likely that in the practical clinical situation the normal colony-forming cells from treated patients will be cycling more rapidly after depletion than in the untreated steady-state, and this could influence their thermal sensitivity because of the differential heat sensitivity among the phases of the cell cycle (Westra & Dewey, 1971).

In view of these clinical considerations, and the lack of knowledge concerning the difference in thermal sensitivity of resting and proliferating normal cell populations, the relative sensitivities of haemopoietic stem cells (CFU-S) and granulocyte-macrophage colony-forming cells (GM-CFC) have been compared using cell populations in different proliferative states. Marrow was taken from untreated mice and also from mice where the marrow was regenerating after a sublethal dose of 4.5 Gy X-rays. The response of fibroblastoid colony-forming cells (CFU-F) in bone marrow was also measured because these stromal cells are transplantable (Piersma *et al.*, 1983), and may contribute to the long-term restitution of haemopoiesis after injury.

### Materials and methods

Female B6D2$F_1$ mice were used throughout at an age of 3 months. Marrow cells were flushed out of the femur with ice-cold Fischer's medium buffered to pH 7.2–7.4 with 20 mM HEPES and containing 20% foetal calf serum. For the study of CFU-F, α-medium was used instead of Fischer's medium. In other experiments with CFU-S and GM-CFC, the serum was omitted because although it is known that serum protects against hyperthermic killing of cell lines maintained *in vitro* (Hahn, 1974; Symonds *et al.*, 1984), there is little knowledge of its protective ability for normal cells tested immediately after extraction from the tissue. A glass bijou bottle containing the cell suspension was shaken and heated in a water bath calibrated at 43±0.1°C. The cell suspension took 3 min to reach 37°C and a further 3 min to reach 43°C, monitored using a thermocouple in conjunction with a digital thermometer. After specified periods of time at this temperature, which was monitored in a dummy replicate, the cell suspension was returned to ice, and assayed shortly thereafter.

Marrow containing regenerating CFU-S and GM-CFC was taken from mice which had received 4.5 Gy to the whole-body 10 days previously, when

*Present address: Department of Radiobiology, Cancer Institute, Chinese Academy of Medical Sciences, Zuo An Men, Beijing, China.
Correspondence: J.H. Hendry.
Received 30 November 1984; and in revised form 7 March 1985.

© The Macmillan Press Ltd., 1985

the doubling times were ~29 h for CFU-S and 24 h for GM-CFC (Testa *et al.*, 1974). With CFU-F, 15 days was chosen as this was the time when the numbers of CFU-F were increasing most rapidly (Xu *et al.*, 1983*a*).

The cycling status of the unperturbed marrow and the regenerating marrow was estimated using the "thymidine-suicide" test, as described previously for CFU-S (Lord *et al.*, 1974), GM-CFC (Iscove *et al.*, 1970) and CFU-F (Castro-Malaspina *et al.*, 1980). Error limits on the levels of kill were calculated as described by Lord *et al.* (1974), together with significance levels (Hazout & Valleron, 1977). After heating, the cell suspensions were assayed, using standard techniques, for surviving CFU-S (Lord & Schofield, 1985), GM-CFC (Testa, 1985) and CFU-F (Xu *et al.*, 1983*b*). The survival curves were fitted using the computer programme described by Gilbert (1969).

### Results

Survival curves for CFU-S, GM-CFC and CFU-F taken from steady-state marrow are shown in Figure 1, and from regenerating marrow in Figure 2. The data were pooled from 2 to 4 experiments. No cytotoxicity was observed for cells kept at 37°C for these times. With the six sets of data shown in Figures 1 and 2, and 4 other sets obtained for cells

**Figure 1** Survival at 43°C of haemopoietic precursors (in medium with serum) in steady-state marrow. Multiple symbols at each time represent replicate experiments. (●) curve S = CFU-S; (■) curve C = GM-CFC; (▲) curve F = CFU-F.

**Figure 2** Survival at 43°C of haemopoietic precursors (in medium with serum) in regenerating marrow. Symbols as in **Figure 1**.

**Table I** Values of $D_0$ for heating at 43°C (standard errors quoted)

| Cell type | [$^3H$]-*TdR kill (%)* | $D_0$ (*minutes*) Without serum | With serum |
|---|---|---|---|
| GM-CFC | 35±3 | 9.7±0.7 | 8.2±0.3 |
| Regenerating GM-CFC | 70±2[a] | 5.4±0.8 | 5.9±0.3 |
| CFU-S | 5±6[b] | 7.4±0.7 | 8.4±0.6 |
| Regenerating CFU-S | 50±3[a] | 6.3±0.9 | 8.7±0.7 |
| CFU-F | 4±4[b] | — | 6.1±0.6 |
| Regenerating CFU-F | 25±3[a] | — | 7.1±0.6 |

[a]Significant increase in cycling compared to the steady state ($P<0.05$).

[b]Not significantly different from zero; all other values significant ($P<0.05$).

heated without serum (Table I), 8 of the 10 extrapolation numbers ranged between 0.9 and 2.2. The other two were 0.3 and 4.0, and out of the 10 only 1 (value 0.3) was significantly different from unity. Thus for simplicity in the comparison of sensitivities, exponential curves with no shoulder were fitted to all the sets of data, and the $D_0$ values are given in Table I.

Mean values of the percentage of the respective cell types killed by thymidine suicide are also shown in Table I. These were measured in a total of 6 experiments which included those where the thermal sensitivity was assessed.

The following points can be noted:

(a) in the steady state, CFU-S and GM-CFC were similarly sensitive, and CFU-F were ~30% more sensitive than the other cell types (i.e. the ratio of $D_0$ values = 6.1/8.3 = 0.7),

(b) there was no consistency in the change in sensitivity with an increase in the cycling rate. GM-CFC become more sensitive by ~45%, but CFU-S and CFU-F did not,

(c) a lack of serum made CFU-S (regenerating or steady-state) ~20% more sensitive, but GM-CFC were little affected.

### Discussion

In normal marrow, CFU-F are slightly more sensitive to heat than the haemopoietic precursors CFU-S and GM-CFC (Table I). This contrasts with X-rays, where the reverse is generally observed (reviewed by Hendry & Lord, 1983). As the S-phase of the cell cycle is a sensitive phase to heat, it was expected that the regenerating cells would be more sensitive. This should apply in particular to CFU-S where the increase in cycling was most marked (Table I). However, the sensitivity was similar with CFU-S in a cycling or a resting state, and the greatest change unexpectedly was with GM-CFC where the increase in thymidine suicide was by only a factor of 2. Serum is known to protect cells against hyperthermic killing (Hahn, 1974) and this was seen in the present work with CFU-S but not with GM-CFC.

A comparison of thermal sensitivities reported in the literature using 43°C is shown in Figure 3. In view of the similarity in sensitivity of steady-state CFU-S and GM-CFC (Table I) it was considered useful to compare the results from different investigators who assayed one or other of the 2 cell types. The range in heating times to achieve a surviving fraction of 0.1 is as much as a factor of 6. This could be due partly to heating at different *absolute* temperatures, because in general a change by 1°C can result in a change by a factor of 2 in the heating time required for a given effect. Two results are included using 42.5°C and 43.5°C, and although the curve for 42.5°C (curve A, Figure 3) demonstrates a sensitivity at the low end of the range reported for 43°C, the curve for 43.5°C (curve D) is in the middle of this range. Thus, as all the investigators used serum, and as changes in the cycling status do not vastly change thermal sensitivity

**Figure 3** Survival at 43°C of CFU-S or GM-CFC (in medium with serum) in steady-state mouse marrow (except where stated).

Curves (A) CFU-S at 42.5°C (Robins *et al.*, 1983); (B) human GM-CFC (Bromer *et al.*, 1982); (C) GM-CFC (van Zant *et al.*, 1983); (D) GM-CFC at 43.5°C (Elkon *et al.*, 1982); (E) CFU-S (Symonds *et al.*, 1984); (F) CFU-S and GM-CFC (present data); (G) CFU-S (Symonds *et al.*, 1981); (×) CFU-S and GM-CFC (Tribukait *et al.*, 1978); (□) human GM-CFC (Tribukait *et al.*, 1978); (△) CFU-S (Elkon *et al.*, 1981); (○) pre-GM-CFC assayed in diffusion chambers (Elkon *et al.*, 1981).

(Table I), other unknown technical factors in the heating technique are likely to be responsible for the remaining differences at 43°C. These may relate to differences in heating-up times and pH.

It should be noted that the survival curve for AKR leukaemic cells (Robins *et al.*, 1983) fell to the left of its companion curve for normal CFU-S (curve A in Figure 3), and it was near the middle of the range of curves shown. The sensitivity of L1210 leukaemic cells (Symonds *et al.*, 1981) was close to the minimum measured for normal CFU-S (curves E to G in Figure 3).

In view of the differences in the sensitivity of normal haemopoietic progenitor cells reported by various investigators, it is clear that if heat is to be used to attempt to kill preferentially any malignant cells in marrow in remission, preliminary experiments to measure the viability of the normal marrow after heating in a particular manner are essential. Erythroid precursors appear to be more sensitive to heat than leukocytic or thrombocytic precursors

(van Zant *et al.*, 1983), but measurements of the sensitivity of GM-CFC – which have a sensitivity similar to that of CFU-S (Table I) – should be sufficient to predict the viability of human marrow in terms of its stem-cell (CFU-S) content, which cannot be assessed directly. Alternatively, the sensitivity of pluripotent progenitor cells (Mix-CFC) could be investigated.

We thank the Cancer Research Campaign (UK) for support.

### References

BROMER, R.H., MITCHELL, J.B. & SOARES, N. (1982). Response of human hematopoietic precursor cells (CFU-C) to hyperthermia and radiation. *Cancer Res.*, **42**, 1261.

CASTRO-MALASPINA, H., GAY, R.E., RESNICK, G. & 6 others. (1980). Characterisation of human bone marrow fibroblast colony-forming cells (CFU-F) and their progeny. *Blood*, **56**, 289.

ELKON, D., SALBI, H., McGRATH, H.E. & BAKER, D.G. (1981). Temperature dependent inhibition of murine granulocyte-monocyte precursors. *Cancer Res.*, **41**, 1912.

ELKON, D., McGRATH, H., CONSTABLE, W. & BAKER, D. (1982). Inhibition of murine hematopoiesis by hyperthermia. *Natl Cancer Inst. Monog.*, **62**, 239.

GILBERT, C.W. (1969). Computer programmes for fitting luck and probit survival curves, *Int. J. Radiat. Biol.*, **16**, 323.

HAHN, G.M. (1974). Metabolic aspects of the role of hyperthermia in mammalian cell inactivation and their possible relevance to cancer treatment. *Cancer Res.*, **34**, 3117.

HAZOUT, S. & VALLERON, A.J. (1977). Planning the suicide experiments. *Cell. Tiss. Kinet.*, **10**, 569.

HENDRY, J.H. & LORD, B.I. (1983). The analysis of the early and late response to cytotoxic insults in the haemopoietic hierarchy. In *Cytotoxic Insult to Tissue: Effects on Cell Lineages*. (Eds. Potten & Hendry). Churchill-Livingstone, Edinburgh, London, Melbourne and New York, 1983, p. 36.

ISCOVE, N.N., TILL, J.E. & McCULLOCH, E.A. (1970). The proliferative status of mouse granulopoietic progenitor cells. *Proc. Soc. Exp. Biol. Med.*, **134**, 33.

LORD, B.I., LAJTHA, L.G. & GIDALI, J. (1974). Measurement of the kinetic status of bone marrow precursor cells. *Cell Tissue Kinet.*, **7**, 505.

LORD, B.I. & SCHOFIELD, R. (1985). Haemopoietic spleen colony-forming units. In *Cell Clones: Manual of Mammalian Cell Techniques* (Eds. Potten & Hendry). Edinburgh: Churchill-Livingstone.

PIERSMA, A.H., PLOEMACHER, R.E. & BROCKBANK, K.G.M. (1983). Transplantation of bone marrow fibroblastoid cells in mice via the intravenous route. *Brit. J. Haematol.*, **54**, 285.

ROBINS, H.I., STEEVES, R.A., CLARK, A.W., MARTIN, P.A., MILLER, K. & DENNIS, W.H. (1983). Differential sensitivity of AKR murine leukaemia and normal bone marrow cells to hyperthermia. *Cancer Res.*, **43**, 4951.

RUBIN, P., WHEELER, K.T., KENG, P.C., GREGORY, P.K. & CROIZAT, H. (1981). The separation of a mixture of bone marrow stem cells from tumor cells: An essential step for autologous bone marrow transplantation. *Int. J. Radiat. Oncol. Biol. Phys.*, **7**, 1405.

SYMONDS, R.P., WHELDON, T.E., CLARKE, B. & BAILEY, G. (1981). A comparison of the response to hyperthermia of murine haemopoietic stem cells (CFU-S) and L1210 leukaemia cells: Enhanced killing of leukaemic cells in the presence of normal marrow cells. *Br. J. Cancer.*, **44**, 682.

SYMONDS, R.P., WHELDON, T.E. & CLARKE, B.M. (1984). Heat sensitivities of murine normal and leukaemic haemopoietic stem cells: Thermal inactivation energy and dependence on nutritional milieu. *Br. J. Radiol.*, **57**, 421.

TESTA, N.G., HENDRY, J.H. & LAJTHA, L.G. (1974). The response of mouse haemopoietic colony forming units to repeated whole-body X-irradiation. *Biomed. Exp.*, **21**, 431.

TESTA, N.G. (1985). Clonal assays for haemopoietic and lymphoid cells *in vitro*. In *Cell Clones: Manual of Mammalian Cell Techniques* (Eds. Potten & Hendry). Edinburgh: Churchill-Livingstone.

TRIBUKAIT, B., SODERSTROM, S. & BERAN, M. (1978). Survival, repair and pH dependence in hyperthermically treated murine and human bone marrow stem cells. In *Cancer Therapy by Hyperthermia and Radiation*. Baltimore/Munich, p. 181.

VAN ZANT, G., FLENTJE, D. & FLENTJE, M. (1983). The effect of hyperthermia on hemopoietic progenitor cells of the mouse. *Radiat. Res.*, **95**, 142.

WESTRA, A. & DEWEY, W.C. (1971). Variation in sensitivity to heat shock during the cell-cycle of Chinese hamster cells *in vitro*. *Int. J. Radiat. Biol.*, **19**, 467.

XU, C.X., HENDRY, J.H. & TESTA, N.G. (1983*a*). The response of stromal progenitor cells in mouse marrow to graded repeated doses of X-rays or neutrons. *Radiat. Res.*, **96**, 82.

XU, C.X., HENDRY, J.H., TESTA, N.G. & ALLEN, T.D. (1983*b*). Stromal colonies from mouse marrow: Characterisation of cell types, optimisation of plating efficiency and its effect on radiosensititivy. *J. Cell Sci.*, **61**, 453.